# Getting Familiar With Biosimilars

**Published:** 2016-04-01

**Authors:** Kelley D. Mayden

**Affiliations:** Wellmont Cancer Institute, Bristol, Virginia

Biotechnology has revolutionized the treatment of many chronic and acute illnesses, including cancer. The latest advance comes in the form of biosimilars. At JADPRO Live at APSHO, Kelley D. Mayden, MSN, FNP, AOCNP®, of Wellmont Cancer Institute, Bristol, Virginia, gave an A-to-Z talk about these drugs and what they mean for the advanced practitioner in oncology."

The science of creating therapeutics in living systems requires us as advanced practitioners to have an understanding of everything from generic drug manufacturing to the global biosimilar market," she said.

## FROM GENERICS TO BIOLOGICS

Drug development is an expensive process, and many of the end products reflect this investment. Traditionally, the development of generic drugs has been essentially as laborious as for the reference product, but with less return on the dollar.

This process changed in 1984, with the passage of the Drug Price Competition and Patent Term Restoration Act, often referred to as the Hatch-Waxman Act. This Act created an abbreviated new drug application for generic approval—the 505 pathway—that eliminated the need for preclinical animal and clinical human drug testing to establish safety. However, a generic product still must prove itself to be bioequivalent with a brand name product.

Generic drugs are small-molecule compounds that are relatively simple. As a result, they can be synthesized with exactness in the laboratory to completely resemble the original compound. The generic must have the same strength, dosage, form, route of administration, and purity of the brand name product; this is not difficult to prove.

Biopharmaceuticals, including biologics, are far more complicated. They include blood and plasma products, vaccines, monoclonal antibodies, cultured cellular and tissue products, and nonrecombinant and recombinant proteins.

Top sellers in oncology in 2013 were bevacizumab (Avastin), pegfilgrastim (Neulasta), rituximab (Rituxan), and trastuzumab (Herceptin). The impending patent expirations for these and some other biologics have triggered interest in developing less expensive compounds akin to generics in this field, termed "biosimilars." These products have the potential to decrease cost and improve access for patients. "Think of a biosimilar as a new version of an existing biologic that has gone off patent," Ms. Mayden said.

Biologics, however, are not the same as generics. They have a complex molecular structure and, unlike generics, they cannot be synthesized to exactness. "They are living, breathing molecules—proteins—that have the potential for variance," she explained.

Contributing to this variance is the potential to produce immunologic reactions, including the triggering of antibodies (which makes the drug less effective) and anaphylactic reactions. "No two drugs or batches of drugs can ever be exactly alike, so we cannot just rubber-stamp them like we do generic drugs," she said.

Ms. Mayden likened the difference between a generic drug and a large biologic to that between a bicycle and a jet airplane. For example, an aspirin contains approximately 21 atoms or 20 daltons and is easy to reproduce, but a large immunoglobulin G antibody is 25,000 atoms or over 20,000 daltons. "Biologics are clearly not generics," she emphasized.

Biosimilars are considered "highly similar" to reference products. Although they may possess some differences in minor inactive components, they cannot demonstrate clinically meaningful differences. They must be the same in terms of safety, purity, and potency.

The Biologics Price Competition and Innovation Act was legislated in 2010 under the Patient Protection and Affordable Care Act. This Act created an abbreviated drug approval process for biosimilars, called the 351 pathway. The first biosimilar in the United States, Zarxio (filgrastim-sndz), launched in 2015.

Although biosimilars are just becoming available in the United States, 19 have already been approved in Europe. "We’ve been able to look to the European experience to gain experience for our own regulatory pathway and the introduction of our first drug," she said. After more than 400 million patient-days of use, no unexpected adverse events have emerged, and European health-care systems have seen a 30% reduction in drug costs (Rovira et al., 2014).

## ESTABLISHING BIOSIMILARITY

"How can you know that a product is biosimilar and that you can use it with your patients?" Ms. Mayden asked. For one thing, she said, there is FDA oversight from the beginning of the drug development process. At the completion of this process, the FDA evaluates "the totality of the evidence" that the compound meets all the requirements of a biosimilar. This comes from analytical studies (structural, functional), animal studies, and human studies. If the structural and functional data are sound, the FDA may eliminate some of the testing to prove biosimilarity.

In humans, investigators evaluate pharmacodynamics, pharmacokinetics, and immunogenicity and do comparative studies. Favorable results on all these processes usually lead to FDA approval, although the FDA can also ask for more data or recommend against approval.

A reference drug may have multiple approved indications. If biosimilarity is proven, the biosimilar may also be approved for these indications, even though it is tested for only one indication. This process clearly saves money for the manufacturer, whose cost savings will ideally be passed along to the patient, explained Ms. Mayden.

When the biosimilar is proven to be exactly like the reference product and to perform like the reference product in every way, only then can it be termed "interchangeable." If it is deemed interchangeable, it can be substituted by the pharmacist without the knowledge of the health-care provider or prescriber.

Not all biosimilars will be deemed interchangeable, and the practice of interchangeability remains controversial and unproven. "I don’t think the FDA is clear on the exact criteria," she said. "Currently, they approach this on a case-by-case basis. There will be controversial issues, and this is something to watch in the days to come."

## IMPLICATIONS FOR ADVANCED PRACTITIONERS

A white paper from 2011 introduced biosimilars from a regulatory, scientific, and patient safety perspective (Zelenetz et al., 2011). Based on a survey of 277 providers, the paper also pointed to a low level of understanding of biosimilars. A 2014 study from Europe, where there has been much more experience with the drugs, indicated that only half of providers have a basic understanding of the drugs (Dolinar & Reilly, 2014).

"So as advanced practitioners, our first responsibility is to become educated," Ms. Mayden said. "We are going to have to make informed decisions about prescribing these products. We will have to ensure that our institutions have protocols in place that prevent drugs from being interchanged when they should not be. We will have to be sure we are monitoring for adverse events and documenting effectively."

There will be a need to educate peers, billing managers, bedside nurses, and patients, and, especially if interchangeability is allowed, collaboration with pharmacists will be critical, she added. Pharmacovigilance—monitoring for and reporting adverse events—will clearly be a responsibility of advanced practitioners.
